# In search of an efficient strategy to monitor disease status of chronic heart failure outpatients: added value of blood biomarkers to clinical assessment

**DOI:** 10.1007/s12471-017-1040-x

**Published:** 2017-10-05

**Authors:** N. van Boven, K. M. Akkerhuis, S. S. Anroedh, L. C. Battes, K. Caliskan, W. Yassi, O. C. Manintveld, J. H. Cornel, A. A. Constantinescu, H. Boersma, V. A. Umans, I. Kardys

**Affiliations:** 1 Department of Cardiology, Noordwest Ziekenhuisgroep, Alkmaar, The Netherlands; 2 Department of Cardiology, Erasmus Medical Centre, Rotterdam, The Netherlands

**Keywords:** Heart failure, Biomarkers, NYHA class, Repeated measurements

## Abstract

**Introduction:**

Blood biomarkers have the potential to monitor the severity of chronic heart failure (CHF). Studies correlating repeated measurements of blood biomarkers with repeatedly assessed New York Heart Association (NYHA) class over a prolonged follow-up period, and concomitantly investigating their associations with clinical endpoints, have not yet been performed.

**Methods:**

Between 2011–2013, 263 CHF patients were included. At inclusion and subsequently every 3 months, we measured N‑terminal pro-B-type natriuretic (NT-proBNP), high-sensitivity troponin T (Hs-TnT) and C‑reactive protein (CRP), and assessed NYHA class. The primary endpoint comprised heart failure hospitalisation, cardiovascular mortality, cardiac transplantation or left ventricular assist device implantation. Time-dependent Cox models were used.

**Results:**

Mean age was 67 ± 13 years, 72% were men and 27% were in NYHA class III–IV. We obtained 886 repeated measures (median 3 [IQR 2–5] per patient). The primary endpoint was reached in 41 patients during a median follow-up of 1.0 [0.6–1.4] year. Repeatedly measured NT-proBNP and Hs-TnT were significantly associated with repeatedly assessed NYHA class, whereas CRP was not (NT-proBNP: β [95% CI]: 1.56 [1.17–2.06]ln(ng/l) increase per point increase in NYHA class, p = 0.002; HsTNT: β [95% CI]: 1.58 [1.21–2.07]). Serially measured NT-proBNP (HR [95% CI]:2.86 [1.73–4.73]), CRP (1.69 [1.21–2.34]) and NYHA class (2.33 [1.51–3.62]) were positively and independently associated with the primary endpoint, whereas Hs-TnT lost statistical significance after multivariable adjustment. A model containing serially measured NYHA class and NT-proBNP displayed a C-index of 0.84, while serially measured NYHA class and CRP showed a C-index of 0.82.

**Conclusion:**

Temporal NT-proBNP, CRP and NYHA class patterns are independently associated with adverse clinical outcome. Serially measured NT-proBNP and NYHA class are best suited for monitoring CHF outpatients.

**Electronic supplementary material:**

The online version of this article (10.1007/s12471-017-1040-x) contains supplementary material, which is available to authorized users.

## Introduction

Adjustment of medicinal treatment for chronic heart failure (CHF) requires considerable clinical acumen and may in some cases cause misjudgement in risk assessment and consequently suboptimal treatment [1–4]. Therefore, several diagnostic tools have been developed over the past decades which aim to objectify disease severity, such as the New York Heart Association (NYHA) functional classification [1, 4], which has limited reproducibility and high inter-observer variability [5]. Conversely, circulating blood biomarkers are less subjective to interpretation, and have the potential to monitor subtle changes in the heart that reflect and possibly predict adverse changes before they become clinically apparent [6]. The use of biomarkers, such as B‑type natriuretic peptides (BNP), cardiac troponins and C‑reactive protein (CRP), for risk stratification of CHF patients has already been demonstrated [7–13]. Moreover, although trials on natriuretic peptide-guided therapy of HF have provided somewhat inconsistent results [9, 14], natriuretic peptide-guided HF therapy has recently been given a class IIa recommendation in American College of Cardiology/American Heart Association HF guidelines to achieve guideline-directed medical therapy [15, 16].

Several studies have previously examined NYHA class in relation to clinical outcome in CHF patients. However, these studies either used single, baseline assessments or two repeated assessments taken in a relatively short time interval [17, 18]. Furthermore, studies on the association between blood biomarkers and NYHA class in CHF are scarce, and studies assessing both these properties repeatedly are non-existent. Finally, the predictive value of serially assessed blood biomarkers and NYHA class scores for adverse clinical outcome has never yet been compared in stable CHF patients.

Therefore, the aim of the current investigation, performed in 263 patients with CHF, was to examine the associations between repeatedly measured NT-proBNP, troponin T (Hs-TnT), CRP, and NYHA class, as well as the associations of their temporal patterns with adverse clinical outcome. Based on this, we evaluated the incremental value of serially measuring blood biomarkers to clinical assessment in terms of serial NYHA class scoring, for monitoring stable CHF outpatients.

## Methods

### Patients

**Fig. 1:**
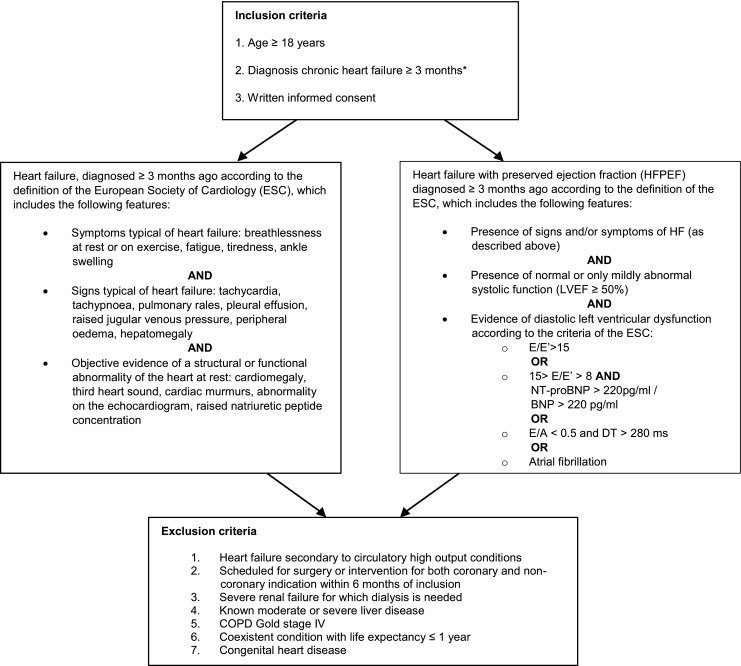
Inclusion and exclusion criteria

The Serial Biomarker Measurements and New Echocardiographic Techniques in Chronic Heart Failure Patients Result in Tailored Prediction of Prognosis (Bio-SHiFT) study was designed to investigate the hypothesis that temporal patterns of biomarkers involved in CHF are associated with prognosis. Bio-SHiFT is an ongoing prospective, observational study of stable outpatients with CHF, conducted in Erasmus MC, Rotterdam, the Netherlands and Noordwest Ziekenhuisgroep, Alkmaar, the Netherlands. Patients were recruited during their regular outpatient visits and were in a clinically stable condition. Patients were eligible for inclusion if aged 18 years or older, capable of understanding and signing informed consent, and if CHF (including HF with preserved ejection fraction) was diagnosed ≥3 months ago according to the guidelines of the European Society of Cardiology (ESC) [1, 4, 19]. Detailed inclusion and exclusion criteria are shown in Fig. 1. The study was approved by the medical ethics committees of the participating hospitals and was conducted in accordance with the Declaration of Helsinki. Written informed consent was obtained from all patients. The study is registered in ClinicalTrials.gov, number NCT01851538. Follow-up for this analysis lasted from October 2011 until November 2013.

### Baseline assessment

At baseline, patients were evaluated by trained research physicians, who collected information on HF-related symptoms, including NYHA class [1, 4]. History of chronic renal failure was defined as a glomerular filtration rate less than 60 ml/min/1.73 m^2^. Alcohol consumption was defined as drinking ≥1 alcoholic consumption per day. Electrocardiography and echocardiography were performed. Data were entered into electronic case report forms. Non-fasting blood and urine samples were collected.

### Follow-up visits

Study follow-up visits were scheduled every 3 months (a window of ±1 month was allowed), for a maximum of 30 months. At each tri-monthly study visit, a short medical evaluation was performed, NYHA functional class was scored and blood and urine samples were collected.

### Blood sampling and biomarker measurement

Blood samples were collected at baseline and at each follow-up visit, and were processed and stored locally at a temperature of −80 °C within 2 h after blood collection. When applicable, samples were transported to the central laboratory (Erasmus MC, Rotterdam, the Netherlands) under controlled conditions (at a temperature of −80 °C), until batch analysis was performed. Thus, the biomarker measurements performed for this study did not lead to treatment adjustments.

Batch analysis of NT-proBNP, Hs-TnT, and CRP was performed in the Clinical Chemistry Laboratory of the Erasmus MC. Plasma NT-proBNP and Hs-TnT were analysed using electrochemiluminesence immunoassays (Roche Diagnostics, Elecsys 2010, Indianapolis, Indiana, USA). For NT-proBNP, concentrations were measured ranging from 0.6 to 4130 pmol/l. Coefficients of variation (CVs) were <5% at mean values ranging from 5.19–274 pmol/l. For Hs-TnT, concentrations were measured ranging from 3–10,000 ng/l. CVs were <5% at mean values ranging from 12.7–1,819 ng/l. CRP was measured using an immunoturbidimetric assay (Roche Hitachi 912 chemistry analyser, Basel, Switzerland). This system measures concentrations ranging from 0.3 to 350 mg/l, and CVs were <5% at mean values ranging from 0.84–284 mg/l.

### Clinical study endpoints

During follow-up, endpoints were recorded in the electronic case report forms by trained research physicians, and associated hospital records and discharge letters were collected. Subsequently, a clinical event committee blinded to the biomarker results reviewed all collected information and adjudicated primary and secondary endpoints.

The primary endpoint comprised the composite of cardiac death, cardiac transplantation, left ventricular assist device implantation, and hospitalisation for the management of acute or worsened HF.

Cardiac death was defined as death from myocardial infarction (MI) or other ischaemic heart disease (ICD-10: I20–I25), death from other heart disease including HF (I30–I45 and I47–I52), sudden cardiac death (I46), sudden death undefined (R96) or unwitnessed or ill-described death (R98, R99). Hospitalisation for acute or worsened HF was defined as exacerbation of symptoms typical of HF, in combination with two of the following: BNP or NT-proBNP > 3x the upper limit of normal, signs of worsening HF, such as pulmonary rales, raised jugular venous pressure or peripheral oedema, increased dose or intravenous administration of diuretics, or administration of positive inotropic agents.

### Statistical analysis

Statistical methods are described in detail in the supplemental text (online Electronic Supplementary Material). In brief, we used linear mixed models to assess the associations between serial biomarker measurements and repeated assessment of NYHA functional class. Associations between serial measurements of biomarkers and NYHA class, and occurrence of the primary endpoint, were examined by entering the serial measurements as time-varying covariates into extended Cox proportional hazards models. First, the models were adjusted for age, gender, systolic blood pressure and estimated glomerular filtration rate (eGFR; calculated using the Chronic Kidney Disease Epidemiology Collaboration (CKD-EPI) equation). Subsequently, all variables, i. e. NT-proBNP, Hs-TnT, CRP and NYHA functional class, were entered simultaneously into the models to investigate their independence. We calculated time-dependent C‑indices based on the extended Cox models. Analyses were performed with R Statistical Software and MedCalc.

## Results

### Study population

A total of 263 patients were included from October 2011 to June 2013. Baseline characteristics are displayed in Tab. 1. Mean age was 67 years (SD ± 13), 72% were men, and 73% were in NYHA class I or II. Median duration of HF at inclusion was 4.6 years (IQR 1.7–9.9). Median baseline NT-proBNP was 137.3 pmol/l (IQR 51.9–272.9), Hs-TnT 18.0 ng/l (IQR 9.6–33.2) and CRP 2.2 mg/l (IQR 0.9–4.8).

**Table 1:** Baseline characteristics

	NYHA class I (*n* = 78)	NYHA class II (*n* = 116)	NYHA class III/IV (*n* = 66)	Total (*n* = 263)
*Demographics*				
Age	64 (±11)	68 (±13)	72 (±11)	67 (±13)
Male gender	60 (78)	81 (69)	48 (70)	189 (72)
Caucasian ethnicity	71 (92)	107 (92)	66 (94)	244 (94)
*Clinical characteristics*				
Body mass index kg/m^2^	28 (±5)	27 (±4)	28 (±4)	28 (±5)
Heart rate, bpm	63 (±10)	68 (±11)	69 (±13)	67 (±12)
SBP, mm Hg	123 (±19)	124 (±20)	120 (±21)	122 (±20)
DBP, mm Hg	75 (±11)	72 (±11)	71 (±10)	72 (±11)
*Biomarker level*				
NT-proBNP (pmol/l)	93 (38–175)	141 (49–583)	225 (120–436)	140 (52–273)
HsTNT (ng/l)	13 (7.8–21)	19 (9.9–37)	24 (16–43)	18 (10–33)
CRP (mg/l)	1.6 (0.6–3.4)	2.3 (1.0–5.3)	2.7 (1.3–4.9)	2.2 (0.9–4.8)
Creatinine (mg/dl)	1.2 (1.0–1.5)	1.1 (1.0–1.4)	1.3 (1.0–1.6)	1.2 (1.0–1.5)
eGFR^a^ (ml/min/1.73 m^2^)	62 (42–83)	58 (46–78)	53 (38–72)	58 (43–76)
*Medical history*				
CAD	29 (39)	57 (50)	56 (84)	142 (46)
ICD	42 (55)	67 (57)	42 (61)	151 (59)
CRT	20 (26)	40 (34)	18 (26)	78 (30)
CVA	8 (10)	18 (15)	15 (22)	41 (16)
Chronic renal failure	34 (44)	61 (52)	41 (59)	136 (53)
Diabetes mellitus	16 (23)	34 (28)	31 (42)	81 (31)
Hypercholesterolaemia	26 (34)	41 (35)	26 (38)	93 (36)
Hypertension	33 (43)	56 (48)	31 (45)	120 (46)
*Intoxications*				
Alcohol consumption	30 (40)	51 (44)	27 (39)	108 (42)
Ever smoker	58 (75)	79 (68)	48 (70)	185 (71)
*Medication use*				
ACE-i or ARB	75 (96)	109 (94)	61 (88)	245 (93)
Aldosterone antagonist	44 (56)	81 (70)	53 (80)	178 (68)
Diuretic	64 (82)	107 (92)	66 (96)	237 (90)
Beta-blocker	68 (87)	106 (91)	58 (88)	232 (88)
Aspirin	14 (18)	16 (14)	15 (22)	45 (17)
Vitamin K antagonist	59 (77)	88 (78)	53 (77)	200 (77)

Normally distributed continuous variables are presented as mean (± standard deviation). Non-normally distributed continuous variables are expressed as median (25^th^–75^th^ percentile). Categorical variables are expressed as count (percentage). Valid percentages are given because of missing values, thus total counts may vary.

*ACE-i* angiotensin converting enzyme inhibitor, *ARB* angiotensin receptor blocker, *CAD* coronary artery disease, *CRP* C-reactive protein, *CRT* cardiac resynchronisation therapy, *CVA* cerebrovascular accident, *DBP* diastolic blood pressure, *eGFR* estimated glomerular filtration rate, *HsTNT* high sensitive cardiac troponin T, *ICD* implantable cardioverter defibrillator, *NT-proBNP* N-terminal pro-B-type natriuretic peptide, *NYHA* New York Heart Association, *SBP* systolic blood pressure

^a^eGFR was calculated using the Chronic Kidney Disease Epidemiology Collaboration (CKD-EPI) equation

### Associations between serial biomarker measurements and serial assessment of NYHA class

During follow-up, we collected 921 blood samples and scored NYHA functional class 1,292 times. Of all follow-up visits, 1,135 took place before the occurrence of the primary endpoint. During these follow-up visits, 886 blood samples were drawn (median 3; IQR 2–5 per patient), and NYHA functional class was scored 1,114 times (median 4; IQR 2–6 per patient).

Repeatedly measured NT-proBNP and Hs-TnT showed strong associations with repeatedly assessed NYHA class, whereas CRP did not (NT-proBNP: β [95% CI]: 1.56 [1.17–2.06]ln(ng/l) increase per point increase in NYHA class, p = 0.002; HsTNT: β [95%CI]: 1.58 [1.21–2.07], p = 0.001; CRP: β [95% CI]: 1.22 [0.98–1.53], p = 0.076) (Tab. 1 of the online Electronic Supplementary Material).

### Clinical endpoints

The composite endpoint was reached by 41 patients (16%), during a median follow-up of 1.0 [0.6–1.4] years: 5 patients died from a cardiovascular cause, 35 patients were re-hospitalised for worsened HF and 1 patient underwent heart transplantation. Of the 35 patients reaching the primary endpoint because of re-hospitalisation for HF, 16 patients died eventually during further follow-up, of whom 12 patients died from a cardiovascular cause. Overall all-cause mortality was 21 (8.0%).

### Baseline biomarker measurements and NYHA class and the primary endpoint

NT-proBNP, Hs-TnT, CRP, and NYHA class all displayed strong and positive associations with the primary endpoint (Tab. 2). After multivariable adjustment, NT-proBNP, CRP and NYHA class remained independently associated with the primary endpoint, while Hs-TnT lost statistical significance. Of all the other baseline characteristics, only age was independently associated with the primary endpoint.

**Table 2:** Associations between blood biomarker measurements, NYHA class, and the primary endpoint

**Associations between baseline measurements and the primary endpoint**				
	*Univariable models* ^*a*^		*Multivariable model* ^*b*^	
	*HR (95% CI)*	*p*	*HR (95% CI)*	*p*
NT-proBNP^c^	3.10 (1.87–5.13)	<0.001	2.37 (1.39–4.02)	0.003
Hs-TnT^c^	1.94 (1.33–2.84)	<0.001	1.50 (0.96–2.33)	0.11
CRP^c^	1.55 (1.12–2.16)	0.005	1.53 (1.10–2.14)	0.013
NYHA class^d^	2.22 (1.41–3.50)	<0.001	2.14 (1.33–3.45)	0.003
**Associations between serial measurements and the primary endpoint**				
	*Univariable models* ^*a*^		*Multivariable model* ^*b*^	
	*HR (95% CI)*	*p*	*HR (95% CI)*	*p*
NT-proBNP^c^	4.02 (2.50–6.47)	<0.001	2.86 (1.73–4.73)	<0.001
Hs-TnT^c^	1.95 (1.36–2.77)	<0.001	1.26 (0.86–1.84)	0.32
CRP^c^	2.08 (1.50–2.87)	<0.001	1.69 (1.21–2.34)	0.001
NYHA class^d^	2.80 (1.83–4.28)	<0.001	2.33 (1.51–3.62)	<0.001

*HR* hazard ratio, *CRP* C-reactive protein, *Hs-TnT* high-sensitive cardiac troponin T, *NT-proBNP* N-terminal pro-B-type natriuretic peptide, *NYHA* New York Heart Association

^a^Including age and gender

^b^Including age, gender, systolic blood pressure and estimated glomerular filtration rate

^c^HR per standard deviation increase in log transformed level

^d^HR per 1-step increase

### Serial measurements of biomarkers and NYHA class, and the primary endpoint

**Fig. 2a–d:**
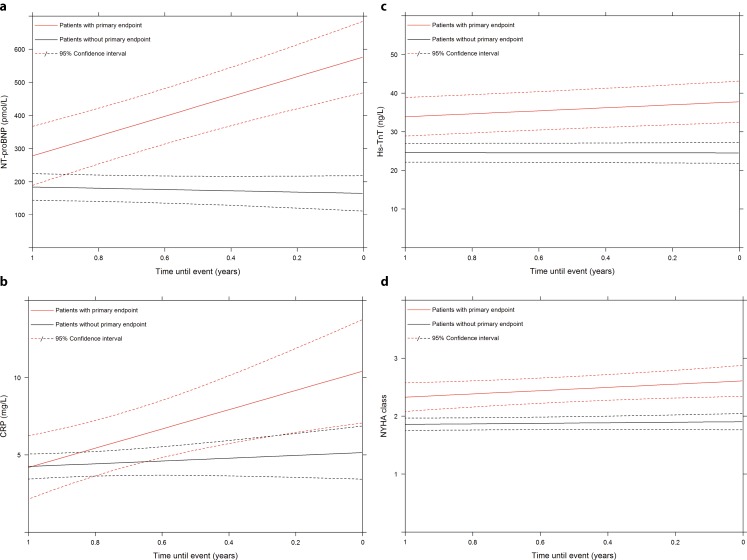
Temporal evolution of serial biomarker measurements and NYHA class. X‑axes display the time that is left until occurrence of the clinical endpoint

Temporal evolutions of serial biomarker measurements and NYHA class are displayed in Fig. 2. Serially measured NT-proBNP, Hs-TnT, CRP, and NYHA class all displayed strong and positive associations with the primary endpoint (Tab. 2). After multivariable adjustment for all serially measured variables, NT-proBNP, CRP and NYHA class remained significantly associated with the primary endpoint.

### Model performance

The discriminative abilities of the models containing baseline measurements of NT-proBNP, Hs-TnT, CRP and NYHA class are shown in the online Electronic Supplementary Material, supplemental Tab. 2, and those of serial measurements in Tab. 3. All individual serially assessed C‑indices, except for Hs-TnT, were numerically higher than corresponding baseline C‑indices. Adding serial NT-proBNP to the model containing age, sex, systolic blood pressure and eGFR and NYHA class provided a substantial increase in C‑index from 0.76 (CI 0.66–0.86) to 0.84 (0.74–0.93), although this did not reach statistical significance (*p* = 0.26). Adding serial CRP instead to this same multivariable model resulted in a C-index of 0.82 (0.72–0.92),* p* = 0.40. Adding both serial NT-proBNP and CRP to the multivariable model only resulted in a slight improvement compared with addition of NT-proBNP only: 0.85 (CI 0.75–0.95), *p* = 0.20.

**Table 3:** Discriminative ability of models containing serial blood biomarker and NYHA assessment

Model	C-index (CI)	P-value
Model^a^	0.62 (0.52–0.71)	NA
Model^a^ + NT-proBNP	0.80 (0.70–0.90)	0.010^b^
Model^a^ + Hs-TnT	0.72 (0.62–0.82)	0.13^b^
Model^a^ + CRP	0.76 (0.66–0.85)	0.048^b^
Model^a^ + NYHA class	0.76 (0.66–0.86)	0.040^b^
Model^a^ + NYHA class + NT-proBNP	0.84 (0.74–0.93)	0.26^c^
Model^a^ + NYHA class + Hs-TnT	0.79 (0.69–0.89)	0.67^c^
Model^a^ + NYHA class + CRP	0.82 (0.72–0.92)	0.40^c^
Model^a^ + NT-proBNP + CRP	0.82 (0.73–0.92)	0.40^c^
Model^a^ + NYHA class + NT-proBNP + CRP	0.85 (0.75–0.95)	0.20^c^

*CI* confidence interval, *CRP* C-reactive protein, *Hs-TnT* high-sensitive cardiac troponin T, *NT-proBNP* N-terminal pro-B-type natriuretic peptide, *NYHA* New York Heart Association

^a^Including age, gender, systolic blood pressure and estimated glomerular filtration rate

^b^P-value compared with model^a^

^c^P-value compared with model^a^ + NYHA class

## Discussion

In this prospective, observational cohort of CHF patients, repeatedly measured NT-proBNP and Hs-TnT were positively and significantly associated with repeatedly assessed NYHA class. Serial assessments of NT-proBNP, CRP and NYHA class were independently associated with adverse clinical outcome. Repeatedly measured NT-proBNP and CRP both added individually to serial NYHA class assessments in terms of discriminative ability. However, a model combining both of these biomarkers with serially scored NYHA class seemed to have little incremental value over serial NYHA class assessment combined with only one of these blood biomarkers. In particular, adding NT-proBNP only seemed the best suited strategy for monitoring stable CHF outpatients.

The strengths of the current study include simultaneous assessment of multiple biomarkers and NYHA class on the one hand, as well as frequent, repeated assessment of these properties on the other hand. Combined with clinical follow-up on adverse events, this renders insight into temporal evolution and manifestation of CHF. On top of that, using biomarker measurements for monitoring patients with CHF has the appealing feature of being objective, and thus uniform and reproducible.

Serial NT-proBNP and CRP measurements were both independently associated with the endpoint and adding serial NT-proBNP and CRP measurements to a model containing NYHA class assessments greatly increased the C‑index, from 0.76 to 0.85. This increase did not reach statistical significance, but recently it has been demonstrated that testing for improvement in prediction performance is actually redundant if a variable has already been shown to be an independent risk factor, and that standard testing procedures such as C‑indices are very conservative and thus insensitive to improvements in prediction performance [20]. Nevertheless, to provide an impression of the magnitude of the incremental prognostic value, we presented C‑indices. Altogether, our results support combining blood biomarkers with clinical assessment for prognostication in CHF patients.

While the prognostic value of blood biomarkers for clinical events has been widely investigated, less is currently known about the association between blood biomarkers and NYHA class in CHF patients. Only two studies have previously assessed this association. These studies measured natriuretic peptide level both at study baseline and at 6 ± 2 weeks of follow-up, and correlated these measurements with, among others, clinical change as categorised by clinicians [17, 18]. Studies performing multiple, repeated measurements of biomarkers and NYHA class over a prolonged follow-up period, and concomitantly investigating their association with clinical endpoints have not yet been performed. Although the NYHA functional classification is a common and globally used system [1, 4], its biggest disadvantage is the non-uniformity in its application by individual clinicians. Raphael et al. conducted a study to investigate consistency in NYHA functional class assessment and found that inter-observer variability was high, with only 54% concordance between two cardiologists [5]. In this respect, adding biomarker information to clinical patient assessment could be valuable for obtaining a more objective estimate of patient prognosis.

### Limitations

Extended Cox models with time-dependent covariates were used to analyse the effects of changes in NYHA class and temporal biomarker patterns on the primary endpoint, because these models are able to accommodate multiple time-varying covariates. However, time-dependent Cox models assume that biomarker levels do not change between measurements [21]. In reality, blood biomarkers are dynamic and continuously change over time, parallel to the condition of the patient. Therefore, we performed a sensitivity analysis by means of a joint modelling approach. Joint models combine a linear mixed-effects model for the serial biomarker measurements with a Cox proportional hazards model for the occurrence of the primary endpoint [21]. We estimated the individual biomarker trajectories and NYHA trajectories using separate joint models, then extracted the fitted trajectories from the joint models, and entered the extracted trajectories simultaneously into one extended Cox model. The results of this analysis were materially the same as those we described in the paper. Furthermore, the majority of the patient population was in NYHA class I or II, and thus at relatively low risk. The results and conclusions should be judged accordingly.

## Conclusions

Serial assessments of NT-proBNP and Hs-TnT are positively associated with NYHA class. Temporal patterns of NT-proBNP, CRP and NYHA class are independently associated with adverse clinical outcome. A model containing these serially measured variables displayed good discriminative ability. However, serially measured CRP had only little incremental discriminative value compared with a strategy combining serial assessments of NYHA class and NT-proBNP. Altogether, our findings underscore the incremental value of biomarkers to NYHA class for monitoring stable CHF outpatients.

## Caption Electronic Supplementary Material

Supplemental text: Full description of the statistical analysis.

Supplemental table 1: Associations between serial blood biomarker measurements and NYHA class

Supplemental table 2: Discriminative ability of models containing baseline blood biomarker- and NYHA assessment
