# Supplementary material for: In search of an efficient strategy to monitor disease status of chronic heart failure outpatients: added value of blood biomarkers to clinical assessment
Source: Neth Heart J. 2017 Oct 5;25(11):634–42. doi: 10.1007/s12471-017-1040-x (PMC5653539; doi:10.1007/s12471-017-1040-x)
Supplement: Supplementary file 1 — Supplemental text: Full description of the statistical analysis. [file 12471_2017_1040_MOESM1_ESM.doc]

**Supplemental text**

**Statistical analysis**

Variables with normal distributions are presented as mean±standard deviation (SD). Variables with non-normal distributions are presented as median and interquartile range (IQR). Categorical data are displayed as count and percentage. In case of skewed distributions, continuous variables were logarithmically transformed for further analyses.

To assess the associations between serial biomarker measurements and repeated assessment of NYHA functional class, we used linear mixed models. Time was used as a random effect. NYHA class was used as the independent variable (fixed effect), in order to be able to uniformly display the change in each of the biomarkers per point increase in NYHA class. Each of the biomarkers was consecutively used as the dependent variable. Associations amongst the 3 biomarkers were examined likewise. For these analyses, all samples drawn were used.

Associations between baseline values of NT-proBNP, Hs-TnT, CRP, and NYHA functional class on the one hand, and the primary endpoint on the other hand, were assessed using only the samples drawn before the occurrence of the primary endpoint. Cox proportional hazards models were used. Associations between serial measurements of the aforementioned variables and occurrence of the primary endpoint were examined by entering the serial measurements into extended Cox proportional hazards models as time-varying covariates. First, the models were adjusted for age, gender, systolic blood pressure and estimated glomerular filtration rate (eGFR; calculated using the Chronic Kidney Disease Epidemiology Collaboration (CKD-EPI) equation). Subsequently, all variables, i.e. NT-proBNP, Hs-TnT, CRP and NYHA functional class, were entered simultaneously into the models to investigate their independence. For serial measurements, this meant that all variables were simultaneously entered as time-varying covariates into the extended Cox analysis. The multivariable models also included age, gender, systolic blood pressure and eGFR.

It has previously been demonstrated that testing for improvement in prediction performance is actually redundant if a variable has already been shown to be an independent risk factor. Independence already proves presence of incremental value [20]. Still, to provide an impression of the *magnitude* of the incremental discriminative ability of the individual and combined serial measurements of the biomarkers and NYHA functional class, we calculated time-dependent C-indices based on the extended Cox models.

Analyses were performed with R Statistical Software using packages ‘Survival’ and ‘nlme’. C-indices were compared using MedCalc. All tests were two-tailed and p-values <0.05 were considered statistically significant.
