# Supplementary material for: In search of an efficient strategy to monitor disease status of chronic heart failure outpatients: added value of blood biomarkers to clinical assessment
Source: Neth Heart J. 2017 Oct 5;25(11):634–42. doi: 10.1007/s12471-017-1040-x (PMC5653539; doi:10.1007/s12471-017-1040-x)
Supplement: Supplementary file 2 — Supplemental table 1: Associations between serial blood biomarker measurements and NYHA class [file 12471_2017_1040_MOESM2_ESM.doc]

**Supplemental table 1 – Associations between serial blood biomarker measurements and NYHA class**

|  | **NT-proBNP^a^** | |  |  |  |  |
| --- | --- | --- | --- | --- | --- | --- |
| **NT-proBNP^a^** | **Β**  **(95%CI)** | **p-value** | **Hs-TnT^a^** | |  |  |
| **Hs-TnT^a^** | 1.66  (1.53 – 1.81) | <0.001 | **Β**  **(95%CI)** | **p-value** | **CRP^a^** | |
| **CRP^a^** | 1.11  (1.06 – 1.16) | <0.001 | 1.03  (0.99 – 1.08) | 0.11 | **Β**  **(95%CI)** | **p-value** |
| **NYHA class^b^** | 1.56  (1.17 – 2.06) | 0.002 | 1.58  (1.21 – 2.07) | 0.001 | 1.22  (0.98 – 1.53) | 0.076 |

CRP = C-reactive protein; Hs-TnT = high sensitive cardiac troponin T; NT-proBNP = N-terminal pro-B-type natriuretic peptide; NYHA = New York Heart Association.

**^a^** Beta coefficient per standard deviation increase in log transformed level

**^b^** Beta coefficient per 1-step increase
