# Supplementary material for: In search of an efficient strategy to monitor disease status of chronic heart failure outpatients: added value of blood biomarkers to clinical assessment
Source: Neth Heart J. 2017 Oct 5;25(11):634–42. doi: 10.1007/s12471-017-1040-x (PMC5653539; doi:10.1007/s12471-017-1040-x)
Supplement: Supplementary file 3 — Supplemental table 2: Discriminative ability of models containing baseline blood biomarker- and NYHA assessment [file 12471_2017_1040_MOESM3_ESM.doc]

**Supplemental table 2 – Discriminative ability of models containing baseline blood biomarker- and NYHA assessment**

| **Model** | **C-index (CI)** |
| --- | --- |
| Model**^a^** | 0.62 (0.52 – 0.71) |
| Model**^a^** + NT-proBNP | 0.76 (0.67 – 0.86) |
| Model**^a^** + Hs-TnT | 0.72 (0.62 – 0.81) |
| Model**^a^** + CRP | 0.68 (0.58 – 0.78) |
| Model**^a^** + NYHA class | 0.71 (0.60 – 0.81) |
| Model**^a^** + NT-proBNP + CRP + Hs-TnT + NYHA class | 0.80 (0.71 – 0.91) |

CI = Confidence interval; CRP = C-reactive protein; Hs-TnT = High-sensitive cardiac troponin T; NT-proBNP = N-terminal pro-B-type natriuretic peptide; NYHA = New York Heart Association.

**^a^** Including age, gender, systolic blood pressure and estimated glomerular filtration rate.
